# Enhanced catharanthine and vindoline production in suspension cultures of *Catharanthus roseus *by ultraviolet-B light

**DOI:** 10.1186/1750-2187-3-9

**Published:** 2008-04-25

**Authors:** Shilpa Ramani, Chelliah Jayabaskaran

**Affiliations:** 1 Department of Biochemistry, Indian Institute of Science, Bangalore – 560012, India

## Abstract

Suspension cultures of *Catharanthus roseus *were used to evaluate ultraviolet-B (UV-B) treatment as an abiotic elicitor of secondary metabolites. A dispersed cell suspension culture from *C. roseus *leaves in late exponential phase and stationary phase were irradiated with UV-B for 5 min. The stationary phase cultures were more responsive to UV-B irradiation than late exponential phase cultures. Catharanthine and vindoline increased 3-fold and 12-fold, respectively, on treatment with a 5-min UV-B irradiation.

## Background

The strong and rapid stimulatory effect of biotic and abiotic elicitors on plant secondary metabolite synthesis attracts now considerable attention and is under intense investigation in many laboratories including ours [1-3]. Chemical treatments such as osmotic shock by mannitol [4], salt stress [5], rare earth elements [5,6] and bioregulators [7,8] have been reported to increase alkaloid production in *C. roseus *cell cultures; however, these increases are cell-line dependent and this greatly limits their utilization [5,9]. Although several approaches have been followed in order to increase the accumulation of alkaloids in cell cultures of *C. roseus*, the yields obtained so far are too low to allow commercial production [7,8]. Efforts are on to look for biotic or abiotic elicitors with more efficient and universal effects on the improvement of indole alkaloids in *C. roseus *cell cultures. Zhao et al. (2000) [10] have reported enhanced production of catharanthine in *C. roseus *cell culture by combined elicitor treatment of an *Aspergillum niger *mycelium and tetramethyl ammonium bromide in shake flasks and bioreactors.

In this study, a suspension culture of *C. roseus *was developed and used as a model system to investigate the effects of UV-B on cell growth, cell viability and secondary metabolite accumulation. It was found that UV-B induced accumulation of catharanthine and vindoline without affecting cell growth and viability.

## Results

### Establishment of a cell suspension culture from leaf explants of *C. roseus*

**Figure 1:**
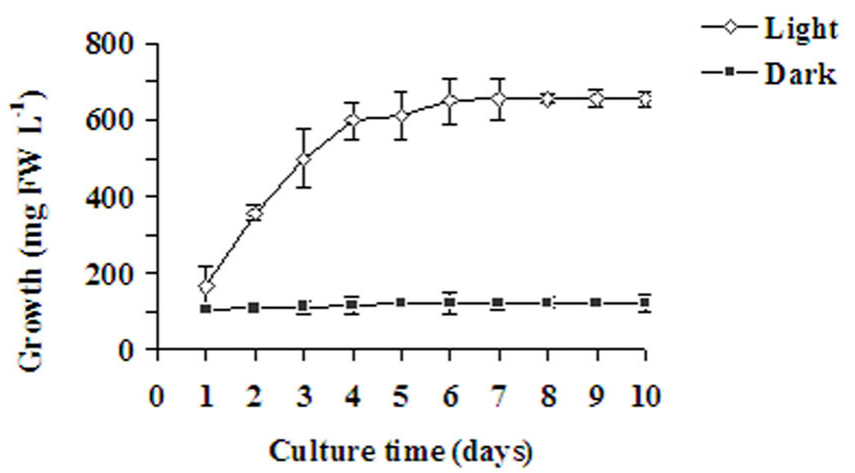
**Time-course of growth of *C. roseus *suspension cultures under light v/s dark conditions**. Actively growing cells from 4-day-old cultures were inoculated into MS medium supplemented with growth regulators and cultured under 16 h day/8 h dark photoperiod or under complete dark conditions. Cells were harvested on the indicated day and the fresh weight was determined. Values are expressed as the means ± SD (n = 3).

Cell suspension cultures were established from the leaf explants of *C. roseus*. These cultures were maintained for three years by sub-culturing at weekly intervals to achieve a homogenous population of cells and used in these studies. Cell growth typically exhibited a rapid growth period between day two to day five, and a stationary phase thereafter (Fig. 1). Cell viability during the growth stage was 93–95% between day four and day six as determined by fluorescein diacetate (FDA) staining (data not shown). Growth of cells increased when the cultures were grown under a 16 h light/8 h dark day/night regime while cells grown in the dark showed no growth (Fig. 1).

### Characteristics of *C. roseus *suspension cultured cells

**Figure 2:**
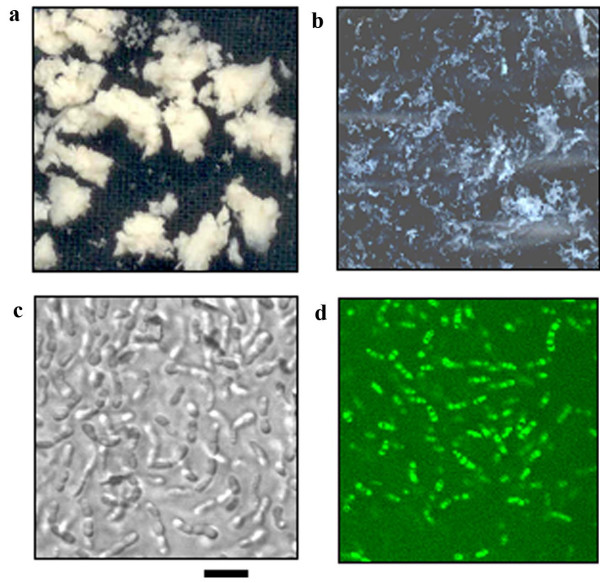
***C. roseus *cell suspension cultures**. ***A***, Compact callus clusters formed after 3 days of inoculation in the MS liquid medium. ***B***, A dispersed cell suspension culture formed after 6 days of inoculation in the above-mentioned medium. ***C***, Microscopic observation of six-day-old culture: arrows indicate divided cells. ***D***, Fluorescence microscopy (495 nm excitation and 520 nm emission) of FDA-stained cells. Six-day-old cultures were used for microscopic observation and FDA staining. Bar represents 10 μm.

The morphological investigation of these established cultures revealed compact callus clusters up to three days after inoculation. The compact callus cluster had a diameter of approximately 3.4 cm, yellowish-green colour and a rough surface of soft quality (Fig. 2a and 2b). The aggregates were highly friable and could be easily disrupted by repeated gentle aspiration and release. After three days, the culture became a dispersed turbid cell culture as callus aggregates formed were released continuously into the medium. The aggregates obtained in the shake flasks increased in their size during growth, and by the sixth day dispersed completely giving rise to fine-textured, pale-yellowish coloured cultures (Fig. 2b).

The cell length/breadth ratio of suspension-cultured cells was on an average 4.67 in both the control and UV-B-irradiated cells. FDA staining and direct microscopic visualization revealed that the cells were cylindrical in shape with a single plane of division (Fig. 2c and 2d). Cells were seen as a chain due to the division being in a single plane. Though the cells appeared like callus, the callus was friable and separated into free cells.

### Effect of UV-B on growth of *C. roseus *suspension-cultured cells

**Figure 3:**
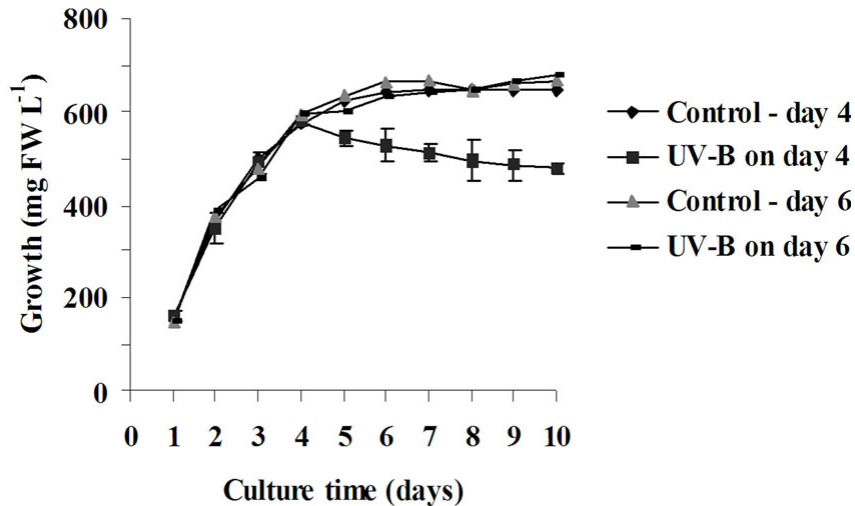
**Effect of UV-B irradiation on growth of *C. roseus *suspension cultures**. Actively growing cells from 4-day-old cultures were inoculated into fresh MS medium supplemented with growth regulators and cultured under 16 h day/8 h dark photoperiod. Cells were irradiated with UV-B on day 4 or day 6 after sub-culture or left untreated as described in materials and methods. Cell growth was measured every day by determining the fresh weight of the harvested cells. Values are expressed as the means ± SD (n = 3).

By the 6^th ^day, the cells had nearly achieved a stationary phase. We investigated if these cells could respond to UV-B. Figure 3 shows that when four-day-old cultures were irradiated for 5 min with UV-B, the fresh weight of the cells decreased gradually. Visualization of these cells by staining with FDA after 24 h of UV-B irradiation showed that 80–90% of these cells were dead. When six-day-old cultures were irradiated with UV-B for 5 min, there was no decrease in fresh weight till 24 h. Furthermore, no significant effect on the cell viability and growth was observed.

### UV-B-induced extracellular medium alkalinization

**Figure 4:**
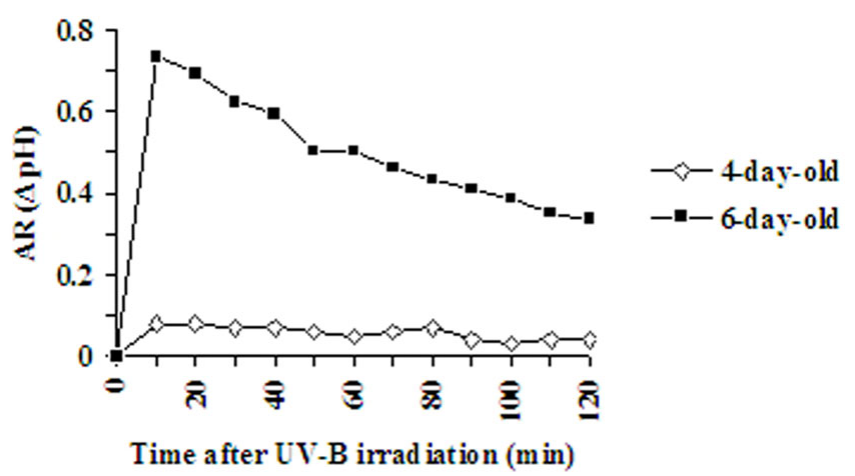
**Medium alkalinization of *C. roseus *suspension cultured cells in response to UV-B irradiation**. Four- and six-day-old cell suspension cultures were irradiated with UV-B for 5 min and the pH of the medium was measured at indicated times after the start of irradiation. Alkalinization Response (AR) or ΔpH was measured as described in materials and methods. Experiments were repeated with two different populations of cells and each experiment was carried out in triplicates.

Four- and six-day-old cell suspensions were exposed to UV-B irradiation for 5 min, and extracellular pH changes were measured in the cell suspension medium for 120 min (Fig. 4). The UV-B-induced AR reached a maximum after 10 min of UV-B irradiation in the six-day-old culture and the increase was about 0.8 pH unit. No significant or only slight pH changes were measured in the four-day-old cultures. These results demonstrate that the four-day-old cultures could not effectively mediate a medium alkalinization response and the six-day-old cultures had gained the ability to exhibit medium alkalinization in response to UV-B irradiation.

### Increased concentration of catharanthine and vindoline on UV-B irradiation

**Figure 5:**
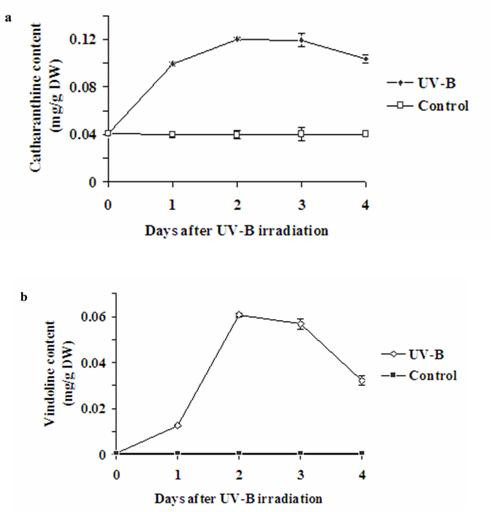
**Time-course of catharanthine and vindoline accumulation in cells irradiated with UV-B in *C. roseus *cell suspension cultures**. Six-day-old suspension cultured cells of *C. roseus *were irradiated with UV-B light for 5 min or left untreated and harvested at indicated times. ***A***, Catharanthine and ***B***, vindoline accumulation was detected by HPLC as described in materials and methods. Values are represented as the means ± SD (n = 3).

Suspension-cultured cells were irradiated with UV-B and the time-course of concentrations of catharanthine and vindoline was followed up to four days after single dose of irradiation by HPLC analysis of the crude TIAs obtained from the cultured cells (Figure 5). Significant differences in catharanthine and vindoline concentrations between UV-B irradiated and the control cells appeared after 24 h. The highest catharanthine/vindoline concentrations were found at 48 to 72 h after UV-B irradiation. As can be seen in Figure 5, the catharanthine and vindoline concentrations in UV-B irradiated cells were significantly higher than those of the untreated controls. The maximum catharanthine and vindoline concentrations obtained in the UV-B irradiated cultures were 0.12 ± 0.0054 mg/g DW and 0.06 ± 0.0023 mg/g DW, respectively, 48 h after UV-B irradiation, while they were 40 ± 0.2 and 0.51 ± 0.3 μg/g DW, respectively, for the control at that time period.

## Discussion

The cell suspension cultures in *C. roseus *have been employed for the production of ajmalicine, catharanthine, serpentine and many other pharmaceutically important products [11]. However, one major disadvantage with the use of cell suspension cultures in *C. roseus *is the inability of the cultures to produce vindoline and hence vinblastine and vincristine [12,13]. In the present study, a cell suspension culture was established directly from the leaves, which produced not only increased amounts of catharanthine but also of vindoline under the UV-B irradiated conditions. The experimental results show that cells at the sixth day were more responsive to UV-B irradiation than cells at day four. The results provide evidence that the UV-B induced medium alkalinization in the cell suspension cultures of 6-day-old cells.

Ouwerkerk et al. (1999) [14] have reported earlier that UV light induces accumulation of several TIAs as well as expression of the TIA biosynthetic genes in excised *C. roseus *leaves obtained from 3-month old greenhouse-grown *C. roseus *plants. In the present study, catharanthine and vindoline concentrations of UV-B irradiated six-day-old culture cells were checked. On the cell dry weight basis, cells irradiated with UV-B produced 2–3 times the amount of catharanthine and vindoline compared to the non-irradiated cells. Catharanthine accumulation in the UV-B irradiated suspension cultures was observed to be 0.12 ± 0.0054 mg/g DW and was comparable to what was reported in *C. roseus *P2 callus cultures (0.12 ± 0.02 mg/g DW) [15]. However, the yield of vindoline in the UV-B irradiated cultures (0.06 ± 0.0023 mg/g DW) was much lower than what has been reported for the P2 callus cultures (0.15 ± 0.02 mg/g DW) [16], but much higher than *A. tumefaciens *transformed suspension cultured cells (~0.017 ± 0.02 mg/g DW) [17].

**Figure 6:**
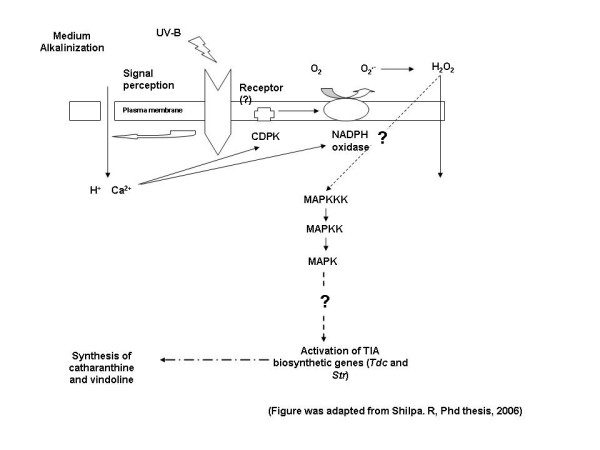
Model for UV-B signaling in *C. roseus*.

In conclusion, the cell suspension culture established has many unique features of growth, behaving both like callus and suspension cultures. Our study also shows, for the first time, the role of UV-B light on enhancement of the concentrations of catharanthine and vindoline in *C. roseus *cell suspension cultures. Figure 6 shows the schematic illustration of UV-B signaling in *C. roseus *suspension cells leading to the increase in catharanthine and vindoline levels. Preception of UV-B induces iron fluxes, medium alkalinization, activation of CDPK and NADPH oxidase. This leads to the activation of ROS production and MAPKinases and eventually stimulates to induce TIA biosynthetic gene expression and the accumulation of catharanthine and vindoline. These findings are of importance for further mechanistic study considering the worldwide demand for catharanthine and vindoline.

## Materials and methods

### Chemicals

Catharanthine and vindoline were obtained from Shanghai Kangai Biologicals, China. Fluorescein diacetate was obtained from Sigma chemical company, St. Louis, USA. All other chemicals were of the highest purity commercially available.

### Establishment of a cell suspension culture, maintenance and culture conditions

Mature leaves were collected from naturally grown plants of *C. roseus *from the campus of Indian Institute of Science, Bangalore. Leaves were cleaned under running tap water, immersed in 0.5% bavistin solution for 20 min and rinsed thoroughly with sterile distilled water. The leaf explants were then treated with 0.01% Tween 20 for 20 min, surface sterilized using an aqueous solution of 0.1% (w/v) mercuric chloride for 5 min, and rinsed extensively with sterile distilled water. The explants were cut into 1 cm^2 ^pieces and inoculated into 200 ml of MS basal medium [18] supplemented with 2 mg/l NAA, 0.2 mg/l kinetin and 3% sucrose in a 500 ml Erlenmeyer flask. The pH of the medium was adjusted to 5.8 prior to autoclaving. The culture flasks were capped with cotton plugs and placed on a gyratory shaker at 60 rpm at 25 ± 2°C with a 16 h photoperiod of 40 μmolm^-2 ^s^-1 ^using cool white fluorescent tubes. After three weeks of culturing, small callus or cell aggregates were released from swollen explants continuously into the medium. The dispersed cell suspension culture so derived was maintained for three years by sub-culturing at weekly intervals to obtain a homogenous population of cells. The cells were sub-cultured every six days, by inoculating 5 mg/l of fresh weight of cells into 500 ml flasks containing 200 ml medium and cultured under the conditions as mentioned earlier.

### Biomass determination

To measure the growth of *C. roseus *cell cultures in shake flasks, fresh weight (FW) of cells was determined by harvesting cells in pre-weighed centrifuge GSI cups from 200 ml culture by centrifugation at 3000 rpm for 15 min. The cups were weighed again, and the fresh weight of the cells was determined as the difference between the total weight and the weight of the empty cup. To determine the dry weight (DW), the cells were harvested as mentioned above and the cells were washed with distilled water, collected, lyophilized and weighed.

### UV-B radiation treatment

For all the experiments involving UV-B treatment, four- (late exponential phase of growth) or six-day-old (stationary phase of growth) cultures were harvested and resuspended in six ml of the culture medium. The remaining medium was saved for subsequent use. Three ml of the suspension culture was used for UV-B irradiation and the remaining three ml was used as a control for the same experiment. After a 1 h equilibration period, the cultures were irradiated for 5 min (three ml in 35 mm open petriplates) with a UV-B lamp (Minera lights, UVM 57, San Gabriel, California) by placing at a distance of 2.5 cms from the lamp source. The intensity of UV-B light on the sample surface was 1.26 μW/cm^2 ^and the total energy supply was 0.34 J/cm^2^. After irradiation, three ml of the suspension cultures (irradiated and control cells) were reconstituted to 100 ml with the saved culture medium in a 250 ml sterile flask and cultured under the same conditions. The cell cultures were harvested at regular intervals and used for further analysis.

### Determination of cell viability, cell morphology and cell aspect (length/breadth) ratio

Fluorescence microscopic analysis was used to distinguish viable (fluorescent) from nonviable (nonfluorescent) cells, following staining with fluorescein diacetate (FDA) by the method of Widholm [19]. Autofluorescence with unstained cells as control was found to be negligible in all cells. To calculate the cell aspect ratio, 200 μl of six -and four-day-old cell suspension cultures (control and UV-B irradiated cells) were taken, stained with FDA (10 μg/ml) for 1 min and viewed using a confocal microscope. The picture of fluoroscein diacetate stained cells was used to measure the length and breadth of each cell to arrive at the average aspect ratio [19]. The cell aspect ratio was measured for 400 cells. To observe the pattern of cell division, late exponential phase cultures were directly mounted on slides and observed at different time points.

### Medium alkalinization response (AR) assay

To determine the UV-B-induced medium alkalinization, 1.5 ml of four- and six-day-old suspension cultures were withdrawn and placed directly in 24-well plates. The cells were equilibrated for a period of 1 h to stabilize the pH of the medium and irradiated with UV-B for 5 min. Extracellular pH changes during growth were measured with pH meter in *C. roseus *cell suspensions. Measurements were carried out for a time-period of 120 min and the experiments were repeated twice in triplicates. UV-B induced medium alkalinization response (AR or Δ pH) was calculated as the increase in pH between the untreated controls and the respective UV-B irradiated samples as described previously [20].

### Terpenoid indole alkaloid extraction and quantification of catharanthine and vindoline by HPLC analysis

The extraction of terpenoid indole alkaloids and quantification of catharanthine and vindoline using HPLC were carried out as described by Schripsema and Verpoorte [21]. Briefly, freeze-dried cells (~50 mg) were extracted twice with 5 ml of dichloromethane, and the extraction solutions were combined and concentrated under vacuum. The residues were dissolved in 0.5 ml of HPLC mobile phase [50 mM sodium phosphate pH 3.9: acetonitrile: 2 methoxyethanol (80:15:5 v/v)] and then analyzed by HPLC using Shimadzu LC-6A instrument with shimadzu CR3A integrator and a SPD-6AV UV detector monitoring at 280 nm. The column was a Nucleosil 5 C18 column (250 × 4.6 mm, 5 μm, Novapak). Identification of compounds was based on the retention time and comparisons of their UV spectra with those of the authentic standards. Amounts of catharanthine and vindoline were finally reported as mg g ^-1 ^DW (dry weight) cells.

## Abbreviations

AR: Alkalinization response; DW: Dry weight; FDA: Fluorescein diacetate; FW: Fresh weight; HPLC: High pressure liquid chromatography; MS: Murashige and Skoog medium; NAA: α- Naphthalene acetic acid; TIA: Terpenoid indole alkaloid; UV-B: Ultraviolet-B light.

## Competing interests

The authors declare that they have no competing interests.

## Authors' contributions

CJ provided project leadership and financial support. Experiments were designed by both the authors and performed by SR. CJ and SR wrote the manuscript, which both the authors read and approved.
